# Automatic case cluster detection using hospital electronic health record data

**DOI:** 10.1093/biomethods/bpad004

**Published:** 2023-03-15

**Authors:** Michael E DeWitt, Thomas F Wierzba

**Affiliations:** Department of Internal Medicine, Section on Infectious Disease, Wake Forest University School of Medicine, Winston-Salem, North Carolina, USA; Department of Internal Medicine, Section on Infectious Disease, Wake Forest University School of Medicine, Winston-Salem, North Carolina, USA

**Keywords:** COVID-19, outbreak detection, public health, epidemiology, cluster detection

## Abstract

Case detection through contact tracing is a key intervention during an infectious disease outbreak. However, contact tracing is an intensive process where a given contact tracer must locate not only confirmed cases but also identify and interview known contacts. Often these data are manually recorded. During emerging outbreaks, the number of contacts could expand rapidly and beyond this, when focused on individual transmission chains, larger patterns may not be identified. Understanding if particular cases can be clustered and linked to a common source can help to prioritize contact tracing effects and understand underlying risk factors for large spreading events. Electronic health records systems are used by the vast majority of private healthcare systems across the USA, providing a potential way to automatically detect outbreaks and connect cases through already collected data. In this analysis, we propose an algorithm to identify case clusters within a community during an infectious disease outbreak using Bayesian probabilistic case linking and explore how this approach could supplement outbreak responses; especially when human contact tracing resources are limited.

## Background

Contact tracing is an effective way to identify transmission chains of infectious diseases and is a well-known tool for public health. From a disease control perspective, the primary goal of contact tracing is to arrest the onward transmission of a pathogen. Through contact tracing, infected individuals are isolated and a history of who an infected individual had close contact with is established. From these interviews, data are generated which allow the creation of the ‘who acquired infection from whom’ (WAIFW), a key component of contact tracing [1]. Individuals with close contact are then interviewed for symptoms, treated as needed, and quarantined to arrest additional transmission of infection. Effective use of contact tracing has been shown to drastically reduce the spread of infection [2]. Additionally, contact tracing can be paired with other interventions such as ring vaccination as demonstrated during Ebola outbreaks where those contacts exposed to an infected individual were prophylactically vaccinated [3].

Traditional contact tracing using public health agents is a time-intensive task and depending on the number of cases and contacts could be a large resource burden. In the case of a reportable disease, public health officials are notified from a variety of sources including clinics and hospitals where a positive test was returned. The staff at the clinic or hospital will then complete a case report form which may be specific to the particular disease and captures information about the patient (e.g. symptoms and demographics) and potential sources of exposure. These forms may be submitted electronically via fax or email to the appropriate public health official for subsequent contact tracing. Importantly, these forms are generally completed as the positive results are returned, so there is a temporal delay between cases which may be related but appear at different times (e.g. due to the incubation period) as well as differences in testing return rates (i.e. the testing delay). However, once the health department has been notified of the reportable disease, contact tracing can begin.

During the severe acute respiratory syndrome coronavirus 2 (SARS-CoV-2) pandemic [4], public health offices were inundated with cases and received guidance to prioritize cases from the Centers for Disease Control & Prevention (CDC) [5]. With each non-isolated positive case potentially making 10 contacts per day [6, 7] coupled with a serial interval of 4–5 days [8], a positive case may make 20–40 unique contacts who would need to be traced. This rough estimate assumes that the infection is detected at the first sign of symptoms, which may not be the case when testing resources are limited or symptoms are initially few. In the context of an epidemic or outbreak with a high number of cases, the vast quantity of contacts that need to be traced can overwhelm public health offices and reduce the likelihood that clusters will be identified. Further underlying the importance of contact tracing and cluster detection, the ability to perform contact tracing in a reasonable amount of time was considered a key part of the CDC’s guide to relaxing restrictions on social distancing in response to the pandemic [9].

An important secondary component of contact tracing is utilizing the data from multiple infections to identify a potential common source. By synthesizing contact tracing data and establishing clusters of cases linked through time, geography, activities, and demography, we can better characterize the underlying risk or behaviors associated with onward transmission. This work is often manually intensive and requires data from multiple cases and contacts to be aggregated and supplemented by additional context. However, by identifying these clusters, targeted resources could be deployed to those in higher risk areas. Additionally, by defining a cluster, contact tracing resources can be prioritized toward those associated with an identified cluster or those cases that are more likely to generate large clusters.

SARS-CoV-2 has been shown to be a virus that can spread in the so-called super-spreader events [10, 11]. This means that one index case may generate many secondary cases, while the majority of transmission stops at a single case [12]. Stopping these super-spreader events is important to ending an epidemic and the spread of infection in a community. Studies have shown that if contact tracing can be used to test, trace, and isolate certain high-risk communities, stopping the spread of infection can be made more effective [13]. As a resource intense operation, contact tracing is often limited by several factors including staffing as well as the openness of the case to name contacts. Providing rapid identification of super-spreading events through the rapid identification of an associated cluster can ameliorate the lack of staffing by focusing resources on known linked cases. Electronic health records (EHRs) programs dominate the private health system with nearly 90% of the office-based physicians reporting the use of EHRs [14]. EHRs have not been adequately explored as an option for rapid contact tracing during an outbreak. Previous research found that the use of EHRs could lead to the identification of additional connected cases [15], but that the process was laborious. EHRs have been used to monitor provider contact with infected persons by using information about which providers went into a particular patient room [16]. While these approaches offer important information in the context of in-hospital infection prevention, it does not immediately address new clusters of cases in the community.

In this study, we explore the concept of automated case cluster detection by leveraging the EHRs and knowledge of the community to augment existing contact tracing operations to ease the burden on contract tracers and more efficiently allocate resources to slow the spread of infection.

## Materials and methods

We aimed to demonstrate the feasibility of using EHR data combined with information about a pathogen to link cases to augment traditional contact tracing methods. We describe the application of the case detection algorithm across a synthetic network in order to explore the properties of the approach.

### Simulation study

We developed a synthetic social network using the *epinet* R package [17] representing geography, households, and primary spoken language. We then modeled the spread of a pathogen across this network in order to create the actual transmission patterns as well as the data that would be available within the EHR. We then utilized the approach as described and implemented in the *autotracer* package (https://github.com/conedatascience/autotracer).

**Figure 1:**
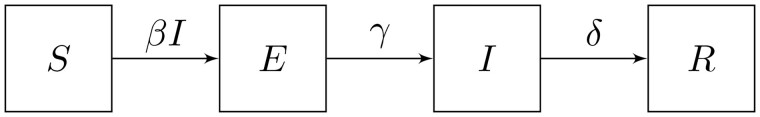
Graphical representation of the SEIR compartmental model.

As described above, understanding the WAIFW matrix is vital for constructing transmission chains and identifying case clusters. In order to generate the WAIFW matrix, the epinet package was used to simulate an outbreak across the synthetic network using a four-compartment model (SEIR) as shown in Fig. 1. In this model, we assume that members of the population are represented in one of the following compartments: susceptible to infection (S), exposed (E), infected (I), or removed (R).

Several key epidemiological parameters govern how the pathogen spreads across the network. In our simulation, we assume a contact rate, β, of 0.2 representing an exponentially distributed 5 days of infection. Furthermore, we assumed gamma distributed latency period (γ, the time spent in the exposed compartment before becoming infected) and recovery periods (δ). In the case of latency, we assumed an average of 0.14 days with a coefficient of variation (CV) of 1.11 and an average of 5.44 days with a CV of 0.18 in the case of recovery. Importantly, these epidemiological parameters should be tuned to the pathogen at hand which is a function of the pathogen and the modes of transmission (e.g. susceptible-infected-susceptible models for some sexually transmitted infections). We can then extract the WAIFW matrix from the output object in order to continue the clustering analysis. We extract the key parameters from our WAIFW matrix and utilize a greedy optimization approach to assign transmission clusters using the algorithm described by Clauset *et al.* [18] and implemented in the igraph package [19]. The average number of secondary infections from this given simulation is roughly 1.69 which approximates the basic reproduction for this pathogen across this network. The time horizon is updated to reflect a date.

### Application of the detection algorithm

The next step in the simulation was to utilize the ‘connect_probable_cases’ from the autotracer package to identify potentially linked cases. This function relies on the outbreaker2 R package [20, 21] which utilizes a Bayesian approach to probabilistically link cases either using the serial interval or generation interval. The ‘connect_probable_cases’ function returns the transmission pair(s) with the highest posterior likelihood of being a transmission event. Full details of the Bayesian models are available in Wallinga and Teunis [22]. Additionally, there is the ability to truncate unlikely transmission pairs. The default value for truncation is taken to be 30 days, indicating that if a transmission pair is greater than 30 days between the recorded cases of the infector and infectee, then it should not be reported as a transmission event is unlikely. Again, this parameter should be tuned based on the epidemiological parameters of the pathogen of interest.

For each scenario, 40 iterations were fitted. For each iteration, a new synthetic population was created, and the spread of infection was simulated across the synthetic social network. The resulting transmission tree was fit with a greedy clustering algorithm [18] and implemented in the R package *igraph* [19] to identify transmission actual ‘clusters’. The transmission tree was then converted to a line list representing the type of data that would be available within an EHR (e.g. patient identifier, information on the household, date of positive test). We then fit the automatic cluster detection algorithm grouping only on the household type and the estimated transmission chain was resulted. For each scenario, the estimated and actual number of clusters and the average cluster size were collected.

### Assessment of the case cluster detection algorithm

In order to understand the performance of the algorithm, we modeled the effect of the average size of the household and the size of the population modeled. We collected a series of summary statistics as shown in the below code which represents the actual number of clusters in our synthetic population versus the number of clusters detected using the algorithm. his simulation process was iterated over a variety of parameters and the root mean squared error (RMSE) was calculated for both the absolute number of clusters as well as the cluster size as shown below:


$$\mathrm{RMSE}\left(\text{Cluster}\;\text{Number}\;\text{or}\;\text{Cluster}\;\text{Size}\right)=\sqrt{\frac{1}{N}\sum_{i}^{2}{\left(y-\hat{y}\right)}^{2}}$$

All analyses were performed using R version 4.1.3 (2022-03-10).

## Results

**Figure 2:**
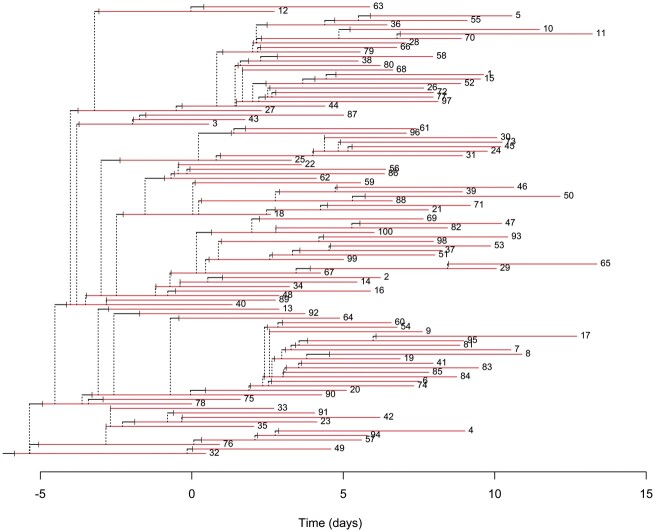
Spread of a pathogen through time in a representative synthetic population. Each horizontal line represents an infected individual. Segments are colored grey when they are exposed and red when they are considered infectious. Onward transmission is represented by vertical dotted gray lines.

**Figure 3:**
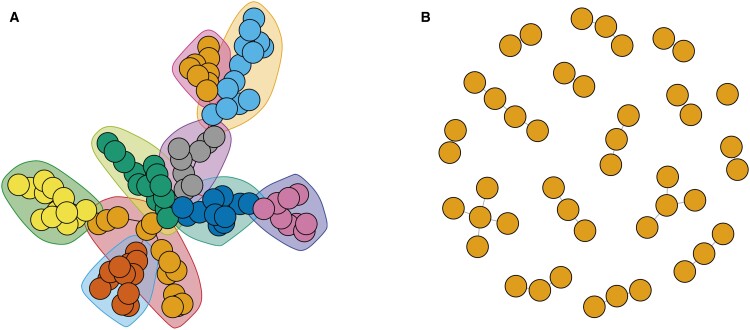
Network diagram of the transmission of a pathogen in a synthetic population with transmission clusters identified through a greedy cluster detection algorithm (A) and inferred cluster network using automatic cluster detection using the automatic cluster detection algorithm (B).

For each simulated iteration, a transmission tree was generated as shown in Fig. 2. Simulated transmission chains were clustered to represent identifiable case outbreaks for each iteration (Fig. 3, panel A). The resulting clusters were then identified using the automatic case cluster detection algorithm (Fig. 3, panel B).

Model performance is shown in Table 1. The automatic cluster detection algorithm tended to overpredict the number of clusters when compared to the actual number of transmission clusters. The number of clusters identified by the algorithm tended to converge with the number of synthetic household units as many of the cases occurred over a rapid timescale. Additionally, the average number of members per transmission chain is underestimated.

**Table 1:** Results of simulation

Average members per household (N)^a^	Population size (N)^b^	Average synthetic clusters (N)	Average estimated clusters (N)	Cluster number error (RMSE)^c^	Cluster size error (RMSE)^c^
4	100	10.3	22.4	12.1	1.2
5	100	10.4	18.3	7.9	0.5
6	100	10.3	15.7	5.5	0.7
5	1000	33.8	196.1	162.3	9.5
5	250	16.5	47.9	31.4	2.7
5	500	23.6	97.7	74.0	5.5

a Model parameter for the average number of members in a household.

b Model parameter for the size of the population in which to simulate the spread of a pathogen.

c Square root of the mean of the squared difference between actual and estimated.

## Discussion

In this analysis, we show that automatic case cluster detection using EHR data could be used as a way of connecting linking cases to identify clusters of cases for more efficient contact tracing resources. While our simulation study used one parameter for group, we were able to clearly identify potential clusters which could be used by public health officials to address possible outbreaks. Furthermore, this study and associated outcomes indicate that the components of the algorithm could be modified in order to fit the social network structure of the local community and the characteristics of the pathogen of interest.

Our simulation appeared to overestimate the number of clusters likely due to our simulation parameterization simulating a single pathogen introduced to a connected network. In a real-world outbreak, there may be multiple introductions of the pathogen rather than a single transmission tree for which we would expect a higher number of clusters, depending on the structure of the social network [23]. In the multiple introduction framework, we would expect the case cluster detection algorithm to better identify these smaller transmission trees.

One of the most important aspects identified through the case cluster detection approach using the EHR is that it is not directly reliant on contact tracing personnel connecting probable cases from a stack of case reports which arrive intermediately. Studies have shown that contact tracing can be effective in reducing the spread of infection, but that relies on contact tracing taking place [2, 24]. Given constrained resources, the use of automatic case clustering can quickly identify which cases are likely linked to a single cluster and thus triage cases from the list of cases that need to be contacted prioritizing labor.

Additionally, there could be additional advantages to the use of a passive cluster detection system. Given the predominance of EHRs, there are opportunities to use automatic case linkage to identify communities which might benefit from potential therapies. For example, as described with ring vaccination, automatic case cluster detection paired with geocoded information may highlight areas that might benefit from targeted recruitment into clinical trials.

It must be noted that other passive surveillance techniques are being explored. These include wastewater surveillance [25] and the use of automated contract tracing utilizing smartphone applications [26]. While wastewater surveillance can provide a directional understanding of the amount of infection within a particular sewer shed, it does not generally provide information necessary for case cluster detection, only presence. Furthermore, studies have shown that individuals may have prolonged fecal shedding of amount of detectable virus, potentially biasing results using wastewater [27]. The use of cellphone-based contact tracing applications requires that participants download and consent to monitor on the individuals’ phones. The application can then signal the individual and/or public health officials regarding those individuals who have had positive contact and trigger the traditional quarantine and support of the contact. The approach relies on participants voluntarily installing and using the contact tracing application which EHR-based case cluster detection does not require. Furthermore, there are unresolved privacy questions including what information is shared (i.e. how much information is stored and retained by the application developers and what information is provided to health departments or provided to other third parties) [15, 28].

### Limitations

It should be noted that this simulation fails to capture the complete nuance of the spread of a pathogen across a social network. The nuance of social contacts within a given community is not well-described [29] and transmission of a respiratory pathogen is fundamentally a stochastic process [30, 31]. While we have attempted to create a realistic scenario to test automatic case cluster detection, demonstration in a community would continue to provide richer data. This includes the chance encounter with an infected individual in a grocery store or any other random connection. Furthermore, people’s behaviors tend to change in the presence of a contagion, making the social network dynamics in the face of disease [32]. However, this simulation demonstrates the ability of automatic case cluster detection to use information that would be slowly pieced together through manual, person-to-person case investigation, and contact tracing in order to identify case clusters. Here using data that are typically reported or available through case report forms, the automatic cluster detection algorithm can probabilistically associate cases into clusters.

Laws such as the Health Insurance Portability and Accountability Act (HIPAA) of 1996 in the USA limit what information can be shared regarding patient health information [33]. As such the deployment of this case cluster detection system may only be applicable to reportable infections and diseases (e.g. COVID-19, human immune immunodeficiency virus, poliovirus, and measles virus) [34, 35]. These are specific diseases for which healthcare providers and other covered entities may disclose protected health information to public health authorities in order to protect the public. This includes information pursuant to conducting public health surveillance, investigations, or interventions. Paramount in the deployment of this algorithm is that these data are available during the case reporting process; importantly, local laws regarding privacy must be observed and may limit what can be automated based on the data that are reported to public health officials. Regardless, automation of case cluster detection may allow public health professionals to better utilize their resources to investigate linked cases and potentially emerging clusters.

Automatic case cluster detection will likely never fully replace the role of human contact tracers and should be viewed as a supplement to human-performed work. The use of the EHR requires that members of the community have access to health care, biasing the case detection toward those with higher levels of socio-economic standing and perhaps more severe disease. This pattern has already been observed in sentinel surveillance programs like ILINet for influenza surveillance where ZIP codes with higher poverty rates were underrepresented [36].

In the case of SARS-CoV-2, the expansion of at-home tests which have not been reported to any health authority has resulted in not only an under-ascertainment of likely infections [37] but also a lost opportunity for contact tracing and cluster detection. While efforts like the USA National Institutes of Health ‘MakeMyTestCount.com’ offer a way for those with positive at-home SARS-CoV-2 tests to report them [38], additional work will be needed in order to integrate these data into public health frameworks such as local health departments. The EHR systems could consider developing a portal in which patients can self-report at-home tests as more and more diagnostics become available for at-home testing for other pathogens such as influenza [39] and respiratory syncytial virus [40]. Data integration exchanges already exist between commercial laboratories and EHRs and could be used as a framework for the integration of at-home test results. For example, the COVID-19 Community Research Partnership utilized at-home lateral flow assays, the results of which were interpreted via an Food and Drug Administration (FDA)-approved smartphone application [41] – a logical extension of which could include data exchange to an authorized EHR or public health authority. It must be acknowledged that there is a tension that exists between testing for clinical use and the regulatory framework designed for clinical validation and testing for surveillance and disease control [42]. While there are technical and legal hurdles that must be overcome (i.e. Clinical Laboratory Improvement Amendments [CLIA] certification), as health monitoring becomes more personal, EHR providers should consider the integration of at-home testing results given their growing role in disease surveillance and public health more broadly.

The use of the EHR makes many strong assumptions. Among these assumptions are that the data available in the EHR are correct. Much of the information about address, language, race, and ethnicity, is manually collected and transcribed into the EHR introducing the opportunity for error. Furthermore, strong assumptions are also made regarding the geocoding of addresses and that people have contacts with those that are collocated with them. For example, a particular address may place two or more people within a particular apartment building, but this does not necessarily mean that transmission occurred between these two people. Place of employment, if limited to employer name alone may not be meaningful for employers with multiple locations (e.g. franchise operations) or for whom employees work remotely. In this case, along with the case of large employers, these linkages may need to be excluded as more likely spurious or paired with additional epidemiological linkages. Additionally, not all cases are identified through testing and available within the EHR which may introduce gaps in estimated transmission chains. Establishing the exact transmission chain is not possible using the EHR information as shown by the performance of our algorithm. As we make strong assumptions about contact patterns using only those data available within the EHR, automatic contract tracing cannot make connections based on unobserved contacts (e.g. social networks, contacts that occur within a doctor’s office or grocery store). Additionally, automatic contract tracing is likely better suited for respiratory infections rather than sexually transmitted diseases as features more important for respiratory pathogens are collected in the EHR than sexual networks.

### Next steps

This methodology, while developed on SARS-CoV-2 and the associated COVID-19 pandemic, could also be used for other infectious diseases within a given community. This approach could surface potential influenza outbreaks as well as other common infectious diseases (e.g. mumps, measles, tuberculosis, gonorrhea, HIV, chlamydia, and Group A streptococcus). Monitoring these infectious diseases and quickly surfacing linked cases would allow the health system and the department of public health to work together to manage outbreaks and develop potential interventions at the earliest possible stages of transmission. However, the automatic cluster detection algorithm should be tuned to the serial interval and routes of transmission of the pathogen of interest. The population of interest could also be tuned such as the use of school districts to highlight potential clusters amongst children or nursing homes for outbreaks amongst the elderly. The use of genetic sequences, when available, could also be employed to further refine estimation of the transmission chains as discussed in Jombert *et al.* [43]. Here the mutational clock and nearest-common ancestor of particular sequences could be paired with epidemiological data in order to inform the inference on the possible linked cases. This would provide a much higher degree of certainty regarding likely transmission but would require that the genetic sequencing information be made available in an EHR and on a timescale that makes intervention possible. However, integration of sequencing information with the EHR when used as a clinical tool requires CLIA certification—a process which requires specific personnel expertise and quality control and assurance procedures [44, 45]. For SARS-CoV-2, while commercial laboratories provided sequencing information, many research laboratories performed sequencing often without CLIA certification [46]. This additional administrative burden can make integration of these data into the EHR prohibitive for contact tracing purposes, especially when sequencing is reliant on research laboratories.

## Implications

### Deploying the methodology in an EHR

In a real-world application, data from the EHR are processed into a local enterprise data warehouse. For a given pathogen, such as SARS-CoV-2, the critical data collected would be the positive test results along with the specimen collection dates. These positive tests could be recorded both for on-site rapid tests and external laboratory results in order to capture the breadth of testing within a community. These positive test results could then be paired with general patient information available within the EHR as part of the normal patient intake process. These data could include the patient’s reported address, their primary spoken language, and employer. Employment history could also be supplemented with insurance information (e.g. plan numbers) if the patient had commercial insurance.

The addresses on file for those patients could then be geocoded using on-premises secure software. Prior to geocoding the addresses, programs could be run to clean the addresses to increase the probability of matching the address in the geocoding server (e.g. consistent abbreviations for streets, removal of apartment numbers, amongst other common misspellings). Geocoded addresses could then be truncated to four decimal places to represent a 30-foot radius around the reported address. A similar approach could be used for employment information with self-reported employment information being cleaned for consistency and compared against a list of locally developed employer aliases (e.g. Proctor & Gamble = Proctor and Gamble) to account for inconsistencies in naming. Consistent cleaning of employers would allow employers to be matched against the North American Industry Classification System provided by the US Bureau of Labor Statistics. Aggregating employers within this framework would provide additional insight as to the types of work where outbreaks are occurring (e.g. manufacturing versus service). Using publicly available data, these cases could be associated with schools given the individual’s age (e.g. an infected child may be zoned to a particular primary school). The primary school could be used as a potential clustering tool and cases could be associated with that school. Given the high number of contacts, increasing the time to identify an emerging cluster in a school could help to stem further spread of infection. While case investigation may arrive at the same conclusion that there is a likely outbreak—the use of the automatic case cluster detection algorithm may reduce the time it takes for this identification to take place.

Using understanding of local demography, a select group of languages could be considered as a potential strong linkage of cultural communities. Because of a strong degree of homophily and the relatively small number of speakers of a given language, connections by language could be invested for potential outbreaks [47, 48].

To establish possible linked cases, the positive patients could be grouped by a given demographic or key metric of interest. These collections of positive respondents would serve as a candidate for a cluster. Once a cluster candidate is identified, a transmission chain could be probabilistically linked using a Bayesian outbreak reconstruction framework and implemented in the R package *outbreaker2* [20, 43]. Literature-reported values could be used for the estimated serial interval [8]. The highest posterior probability transmission chain could then be taken as the most likely transmission chain. This process could then be repeated for employers, language groups, and persons in large congregate settings (e.g. migrant housing communities and trailer parks). If the time between cases was greater than a reasonable threshold given the observed serial interval (e.g. 30 days in the case of SARS-CoV-2), then the transmission chain could be truncated.

The derived cluster information could then be combined from the three different sources and paired with other demographic information like age, race, and gender. If an individual appears in more than one contact network, the earliest known transmission chain could be retained. These resulting data could be then combined into a line list or list of all positive cases, and a contact list that contained the above information regarding who likely was the index case for each infection and identify the common suspected etiology of the cluster.

Automatic cluster emergence can further be enhanced by running this automatic case cluster detection algorithm each day and writing out the cluster identifiers and associated members to a file. The cluster sizes and associated members are recorded each day in order to identify which clusters are growing. Another approach would be to run this algorithm iteratively, sub-setting the data on date (e.g. run the algorithm for all cases less than date n_t_, then repeat for date n_t+1_, and so on. Emerging clusters could then be identified in memory; however, running this algorithm in such a way would likely be computationally costly.) Additionally, the tools provided by the epi-contacts package allow for secondary analysis such as establishing contact patterns and estimating the over-dispersion of the transmission chains using the features of the cases (e.g. age and other demographics).

### Real-world application

This automatic cluster detection algorithm was deployed in the field at a regional health system as described in DeWitt *et al.* [49]. When applied in this context, the clustering algorithm was able to detect an emerging cluster of SARS-CoV-2 infections within a unique cultural group within the local community. Previously, positive cases had been reported incrementally as a function of the incubation period of the disease amongst the positive contacts and the delayed distribution in the return of tests. This delay introduced a substantial time delay between cases of the same cluster. Furthermore, the high incidence of cases in the community masked this emerging cluster was not readily identified through normal contact tracing approaches. However, through the application of the case cluster detection algorithm, this growing cluster was rapidly identified as a probable cluster automatically. The identification of this cluster led to the deployment of additional targeted public health resources including community educators and a mobile testing unit in order to address this rapidly identified case cluster. Targeted mobile testing led to the identification of additional infections and the need for additional resources and education to isolate infection. Exact details cannot be disclosed due to the sensitive nature of the outbreak and to not stigmatize the local community.

When deployed in an actual epidemic, the clustering algorithm successfully identified a series of connected cases within a particular cultural community. This led to the deployment of targeted resources leading to additional identification of cases, education on disease transmission, and support of isolation and quarantine measures.

## Conclusion

Automatic case cluster detection using the EHR provides a methodology to probabilistically identify transmission chains and likely emerging clusters of cases. During an outbreak, when the burden of contact tracing falls on local departments, the use of such tools could help focus limited time and resources during emerging outbreaks. Automatic case cluster detection should be viewed as a complement to contact tracing done by human. By adding a level of automation and providing a higher-level view of possible linked cases, general public health policy and practice could be informed. The use of automatic contract tracing could be used conditionally based on the pathogen or on the case burden within a given community. Furthermore, consideration must be taken on the tradeoffs between false positives and faster identification of potential outbreaks using automatic contract tracing in any context.

## Availability of data and materials

All code and materials used in this manuscript will be upon publication available at: https://github.com/medewitt/network-simulations.

## Authors’ contributions

Michael DeWitt (Conceptualization [lead], Formal analysis [lead], Investigation [lead], Methodology [lead], Software [lead], Validation [equal], Visualization [lead], Writing – original draft [lead], Writing – review and editing [lead]) and Thomas Wierzba (Conceptualization [equal], Formal analysis [supporting], Investigation [supporting], Project administration [equal], Supervision [lead], Validation [supporting], Writing – review and editing [equal]). All authors contributed to the article and approved the submitted version. M.D. authored the algorithms, performed the analysis, and wrote the initial draft. Thomas Wierzba assisted in designing the study, interpreting results, and provided insight into applications for the findings. Both authors edited the final manuscript.
